# Engineering MSC-Derived Small Extracellular Vesicles for Targeted Cargo Delivery to the Injured Spinal Cord

**DOI:** 10.3390/ijms27188308

**Published:** 2026-09-18

**Authors:** Masahito Nakazaki

**Affiliations:** 1 Department of Neural Regenerative Medicine, Institute of Regenerative Medicine, Sapporo Medical University School of Medicine, Sapporo 060-8556, Japan; masahito.nakazaki@yale.edu; 2 Department of Neurology, Yale University School of Medicine, New Haven, CT 06510, USA

**Keywords:** spinal cord injury, small extracellular vesicles, targeted delivery, surface engineering, cargo loading, mesenchymal stem cells, blood–spinal cord barrier, microRNA, drug delivery

## Abstract

Mesenchymal stromal/stem cell-derived small extracellular vesicles (MSC-sEVs) improve locomotor outcomes in preclinical models of spinal cord injury (SCI). After systemic administration, however, most of the MSC-sEVs are taken up by the liver, spleen and lungs, so only a small fraction reaches the injured cord; the vesicle surface and contents are determined by an unmodified parent cell, so both recipient cell engagement and cargo delivery remain heterogeneous rather than specified. Surface engineering and cargo loading may address these limitations. Because targeting and cargo determine the outcome together in vivo, the two are treated here as one framework. The extent to which these strategies have been combined in MSC-sEVs has not been established. This review identifies which MSC-sEVs tested in the injured cord display an engineered ligand, which carry a deliberately loaded cargo, and which do both. A target × cargo × engineering method matrix records the model and carrier of each entry. It shows that an engineered ligand has been paired with a deliberately loaded cargo in two studies on an MSC-sEV tested in SCI, but that neither reports the ligand density per particle or the delivered fraction of the dose, so the contribution of the combination remains unmeasured.

## 1. Introduction

### 1.1. The Delivery Problem in Spinal Cord Injury

Traumatic spinal cord injury (SCI) produces lasting motor, sensory and autonomic deficits. The injury evolves in two phases. The primary mechanical insult disrupts axons, blood vessels and cell membranes within seconds, and a protracted secondary phase follows over hours to weeks, during which ischemia, excitotoxicity, oxidative stress and inflammation expand the lesion well beyond its original margins [1]. This secondary cascade, rather than the primary impact, accounts for most of the eventual tissue loss, and it defines the therapeutic window within which any intervention must act.

A central feature of this cascade is disruption of the blood–spinal cord barrier (BSCB), the highly selective interface formed by endothelial tight junctions, pericytes and astrocyte end-feet that normally isolates the cord parenchyma from the circulation [2,3]. After injury, the BSCB breaks down, allowing infiltration of peripheral immune cells and serum proteins and amplifying inflammation, yet the same barrier later reseals and excludes most systemically administered drugs from the lesion. Inflammation itself follows a defined temporal and cellular sequence, with sequential recruitment of neutrophils, monocyte-derived macrophages and resident microglia that together shape whether the lesion resolves or expands [4]. The injured cord therefore presents a moving target: a microenvironment whose composition, accessibility and dominant cell populations change from the acute to the chronic phase.

This evolving pathology requires interventions that address multiple processes, including inflammation, tissue loss and BSCB repair. Mesenchymal stromal/stem cells (MSCs) are a promising approach to meeting this need. Intravenous infusion of MSCs improves functional recovery in acute, subacute and chronic models and has been used in patients [5,6,7]. However, the infused cells show low and transient engraftment, indicating that much of the benefit results from secreted factors that act on multiple cellular and molecular targets.

Among these secreted products, MSC-derived small extracellular vesicles (MSC-sEVs) are nanoscale membrane-bound particles that carry proteins, lipids and nucleic acids from their parent cells. Intravenously infused MSC-sEVs home to the injured cord, accumulate at the lesion and selectively interact with CD206-positive macrophages [8], where they promote an anti-inflammatory phenotype, stabilize the microvasculature and improve motor recovery [9]. Subsequent work showed that systemically delivered MSC-sEVs improve body growth and motor function after severe injury [10], contribute to BSCB repair [11] and, when infused continuously, reduce scar-forming extracellular matrix (ECM) deposition at the lesion, achieving greater functional recovery than repeated bolus injections [12]. These vesicles were enriched in fibrosis-related microRNAs, suggesting that their microRNA cargo contributed to ECM remodeling [12]. MSC-sEVs thus combine two properties that the delivery problem demands: they reach the injured cord, and they carry a molecular payload to the cells that matter.

Of the administered dose, however, only a small fraction of the administered dose reaches the injured cord, whereas most is taken up by off-target organs. The surface ligands and molecular cargoes of unmodified sEVs are determined by the parent cell, resulting in heterogeneous biodistribution, recipient-cell engagement and cargo delivery. Thus, neither cellular specificity nor the delivered cargoes can be precisely controlled. These limitations have motivated two complementary strategies: targeting engineering to modify biodistribution and cellular engagement, and payload engineering to control cargo identity and loading.

### 1.2. Rationale and Question of This Review

Surface engineering and cargo loading have largely been investigated independently, and previous reviews follow the same division, treating EV delivery platforms, surface targeting and cargo loading as separate subjects. Even the review that most closely addresses both presents them in separate tables [13]. At the particle level, however, the two strategies must be considered together because surface targeting does not control cargo, and cargo loading does not control biodistribution or recipient-cell engagement. In vivo, the outcome reflects both the amount that reaches the cord and what it delivers there, so the contribution of either cannot be evaluated without the other. However, it remains unclear which MSC-sEVs evaluated in SCI display an engineered ligand, carry a deliberately loaded cargo or combine both modifications. The present review addresses this question using a target × cargo × engineering method matrix (Table 1), with each entry classified by experimental model and carrier type. This framework distinguishes constructs evaluated in SCI from strategies demonstrated using other carriers and identifies combinations for future testing.

## 2. MSC-Derived Small Extracellular Vesicles as Delivery Vehicles and Why They Are Engineered

### 2.1. Parent Cell and Tissue Source

Mesenchymal stromal/stem cells (MSCs) are multipotent stromal cells whose therapeutic effects are attributed largely to secreted factors rather than sustained engraftment. MSC-sEVs are one component of these secreted factors and act together with soluble mediators; however, their relative contribution to the therapeutic effects of MSCs in SCI has not been quantified. Among EV-based therapeutic platforms, MSC-derived EVs are the most extensively developed. A 2023 survey found that MSC-derived EVs accounted for 49 of 73 registered therapeutic EV trials [35]. They are also the only EV source with scientific-society recommendations for definition [36] and potency testing [37] and with a translational agenda described in society syntheses [38].

The MSCs used to produce therapeutic vesicles are drawn from several tissues, including bone marrow, umbilical cord, adipose tissue and amnion, and whether the resulting vesicles are interchangeable is unresolved. Two preclinical syntheses have examined source effects. A network meta-analysis of 40 rat studies [39] and a meta-analysis of 21 studies [40] both found no statistically significant difference in motor outcomes between sources, but the comparisons were unbalanced or indirect, the preparations were heterogeneous, and both detected evidence of publication bias. Direct molecular comparisons point away from equivalence: adipose, bone marrow and amniotic membrane MSCs share a conserved core of anti-inflammatory and matrix-regulatory factors yet differ clearly in EV-miRNA content [41], and EVs from bone marrow, umbilical cord and adipose MSCs suppress cytokine release inconsistently across sources in microglia and splenocytes [42]. The source therefore remains an unresolved variable rather than a settled equivalence, and potency must be defined by assay rather than by tissue of origin (Section 6).

### 2.2. Nomenclature

Extracellular vesicles are a heterogeneous population of membrane-bound particles released by virtually all cell types, and the small subset (sEVs, roughly 30 to 200 nm) carries proteins, lipids and nucleic acids that reflect and transmit the functional state of the parent cell. Because the field has used inconsistent nomenclature, this review follows the operational definitions and reporting standards set out in the Minimal Information for Studies of Extracellular Vesicles guidelines (MISEV2023), which recommends the generic term extracellular vesicle, defined by physical and biochemical criteria rather than by presumed biogenesis, and discourages the term exosome unless endosomal origin is demonstrated [15]. Accordingly, the term MSC-sEVs denotes vesicles whose mesenchymal stromal/stem cell parent is established, and the term sEVs is used where the source is generic. The broader term extracellular vesicle is used when a cited study did not restrict its preparation to small vesicles.

Previous reviews have examined individual components of EV engineering. General reviews describe EVs as drug delivery platforms [43,44], natural and engineered targeting [45], parent cell engineering and post-isolation functionalization [46], surface modification [47] and cargo loading and scale-up [48]. SCI-focused reviews address EV biology and microRNA cargoes [49], EV–biomaterial systems [50], engineering strategies [51] and MSC-derived exosomes as drug carriers [52]. Few of these consider surface targeting and cargo loading together in MSC-sEVs for SCI.

### 2.3. Properties and Limits

MSC-sEVs carry the therapeutic content of a cell without the risks of transplanting one. Unlike their parent cells, they are not confined to the vasculature, and MSC-sEV-associated tracers are detected in the injured cord after systemic administration [8,9]. Their lipid bilayer can protect labile cargoes such as RNA from extracellular degradation, although comparative pharmacokinetic data against free molecules are limited. As acellular products, MSC-sEVs avoid the risks of cell replication and ectopic differentiation that are associated with transplanted cells, and they are reported to be poorly immunogenic [53,54,55], although the immunogenicity of engineered products requires product-specific testing. Finally, MSC-sEVs are tractable as an engineering platform: their surface can be decorated with targeting ligands, and their interior can be loaded with therapeutic molecules, which converts a naturally homing particle into a programmable carrier [56].

Unmodified MSC-sEVs have two major limitations: low delivery to the injured cord and limited control over their surface and cargo composition. The first limitation concerns biodistribution. After systemic infusion, MSC-sEVs are distributed throughout the body and are rapidly taken up by the liver, spleen and lungs. Consequently, only a small fraction of the administered dose reaches the spinal cord [57,58,59]. Although EVs cross the blood–brain barrier at measurable rates [60], evidence for entry into the injured spinal cord is based on lesion accumulation rather than direct measurement of influx. The fraction of the administered dose that reaches the lesion site therefore remains undetermined. Thus, the principal limitation is not the ability of MSC-sEVs to enter the injured cord, but the low delivery efficiency relative to uptake by off-target organs.

The second limitation is the lack of control over surface and cargo composition. Unmodified MSC-sEVs display ligands expressed by the parent cell and contain cargoes packaged by that cell. Consequently, the specificity of cellular engagement and the amount of each effector delivered cannot be precisely controlled. The isolation method and the source and state of the parent cells further affect vesicle composition and potency, complicating dose definition and comparisons among studies. Surface display of a targeting ligand increases engagement with the intended cells, whereas deliberate cargo loading controls the identity and amount of the delivered cargoes. Both modifications are required because surface targeting does not control cargo composition, and cargo loading does not redirect the native biodistribution of the vesicles.

## 3. Targeting: Building and Routing the Vesicle to Its Target

**Figure 1:**
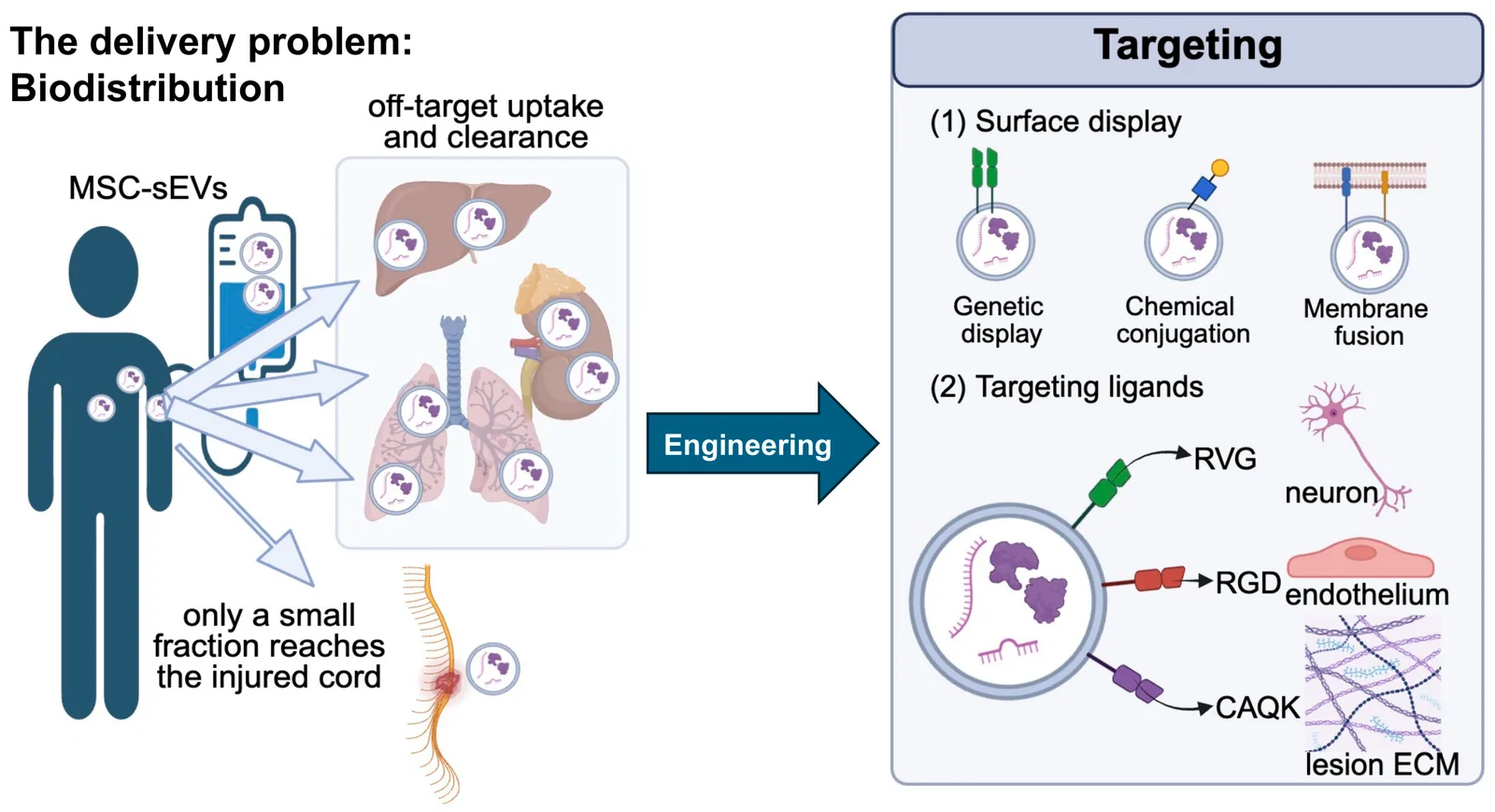
The delivery problem and targeting of engineered MSC-derived small extracellular vesicles for the injured spinal cord. The core obstacle is biodistribution (**left**): administered intravenously, mesenchymal stem cell-derived small extracellular vesicles (MSC-sEVs) spread throughout the body and are lost mainly to off-target uptake and clearance, so only a small fraction reaches the injured cord. Engineering improves targeting (**right**), the set of properties that determine where vesicles accumulate and which cells they engage with. Targeting ligands are incorporated on the vesicle surface via genetic display, chemical conjugation or membrane fusion, and SCI-relevant ligands direct vesicles to distinct destinations: RVG to neurons, RGD to the neovascular endothelium and CAQK to the lesion extracellular matrix. The second engineering axis, the payload (the cargo and how it is loaded) and the full target × cargo × engineering method matrix are examined in Section 4 and Section 5 and Table 1. Abbreviations: CAQK, a lesion-homing peptide (Cys-Ala-Gln-Lys); ECM, extracellular matrix; MSC-sEVs, mesenchymal stem cell-derived small extracellular vesicles; RGD, the Arg-Gly-Asp integrin-binding peptide; RVG, rabies virus glycoprotein-derived peptide; SCI, spinal cord injury. Created in BioRender. Nakazaki, M. (2026) https://BioRender.com/f2mu00v.

Targeting determines where vesicles accumulate and which cells they interact with (Figure 1). It depends on properties derived from the parent cell, modifications introduced through engineering and how the vesicles are administered.

### 3.1. Natural Lesion Accumulation

Unmodified MSC-sEVs reach the injured cord without any engineering, which establishes the baseline that surface modification seeks to improve. After intravenous infusion in rodent models of SCI, MSC-sEV-associated signals were enriched at the lesion relative to the intact cord and were detected in CD206-positive lesion macrophages [8,61]. There they promote an anti-inflammatory macrophage phenotype, upregulate TGF-β, stabilize the microvasculature and improve motor recovery [9]. Systemically delivered MSC-sEVs improve body growth and locomotion after severe injury [10], and amnion-derived MSC-sEVs suppress harmful neutrophil extracellular trap (NET) formation at the lesion through exosomal miR-125a-3p [19].

Most in vivo biodistribution data, including the reported accumulation of MSC-sEV signals in macrophages at the lesion site [8], were obtained using lipophilic membrane dyes. The extent of labeling artifacts depends on the dye and labeling protocol. With the ultracentrifugation-based labeling protocol, approximately nine of every ten PKH26-positive particles are dye nanoparticles rather than labeled vesicles. Recipient cells internalize these nanoparticles into the same compartments as labeled vesicles [62]. Lipophilic fluorophores also desorb onto lipoproteins and self-assemble into vesicle-like particles in serum [63]. Genetic reporters have limitations because the NanoLuc-CD63 fusion itself alters biodistribution. Radiolabeling remains the most accurate method for quantifying biodistribution [64]. No available dye-based method avoids these artifacts. Thus, colocalization of a dye with a cell type provides limited evidence of vesicle delivery.

Two features of the injured spinal cord explain lesion accumulation. First, BSCB disruption during secondary injury allows circulating vesicles to enter the spinal cord [2,3]. Second, the lesion contains inflamed endothelium and abundant phagocytic macrophages. Rapid phagocytic uptake preferentially retains vesicles in macrophages that drive secondary damage. This accumulation is consistent with passive entry through the disrupted barrier followed by phagocytic sequestration. The contribution of ligand-mediated homing has not been isolated experimentally. MSC-sEVs accumulate at the lesion site and have therapeutic value, but two limitations remain. Following systemic infusion, most of the administered dose is distributed to the liver, spleen and lungs [57]. In addition, investigators cannot control which cells take up the vesicles. Surface engineering addresses these limitations by increasing the fraction of the dose that reaches the spinal cord and improving selectivity for the intended cells.

### 3.2. Surface Engineering for Active Targeting

Surface engineering incorporates defined ligands on the vesicle membrane so that a larger fraction of the dose reaches the cord and engages with a specific cell type. Approaches differ in how the ligand is attached, which determines the trade-offs among specificity, yield, stability and manufacturing complexity. Regardless of the engineering method, protein adsorption alters the vesicle surface presented to recipient cells. Proteins from culture medium and serum form a corona on MSC-EVs that controls cellular uptake and in vivo kinetics. Albumin binding alone is sufficient to redirect vesicles away from hepatic macrophages [59]. Thus, displayed ligands compete with an adsorbed protein layer that is neither designed nor routinely characterized. Targeting improvements are almost always reported as fold changes relative to an untargeted control, rather than as the fraction of the injected dose that reaches the spinal cord. This reporting approach prevents comparisons of the practical benefit of targeting ligands across studies.

#### 3.2.1. Genetic Display of Targeting Ligands

In genetic display, the parent cell is engineered to express a targeting peptide fused to an abundant vesicle membrane protein so that the peptide is displayed on secreted vesicles. The strategy was established with the neuron-targeting rabies virus glycoprotein (RVG) peptide fused to the vesicular protein Lamp2b in dendritic cells; the secreted vesicles were loaded with siRNA, and intravenous delivery produced specific, non-immunogenic knockdown of a therapeutic target in the mouse brain [21]. The parent cell there was a dendritic cell, but the same genetic display strategy applies directly to MSCs and their sEVs. In an in vitro blood–brain barrier model, the same RVG-Lamp2b construct directed vesicles through transcytosis rather than through nonspecific uptake [65]. The displayed ligand therefore raises the fraction that reaches neurons rather than granting access that native vesicles lack. Genetic display provides stable expression at the level of the producer cell and accommodates peptide ligands, but the fraction of ligand-positive vesicles and the ligand density per particle remain heterogeneous and can be resolved only by single-particle characterization (Section 6.1). The method also requires engineering of the parent cell and is confined to ligands that tolerate fusion and intracellular trafficking.

#### 3.2.2. Chemical Conjugation and Lipid Anchoring

Chemical conjugation attaches ligands to vesicles after isolation, which decouples targeting from parent cell engineering and admits ligands that genetic fusion cannot accommodate, including antibodies, small molecules and synthetic moieties. Ligands are coupled covalently to surface proteins through amine-reactive chemistries, inserted into the membrane on lipid anchors, or attached via bio-orthogonal click chemistry, which joins pre-functionalized vesicles and ligands chemoselectively and in high yield under mild conditions [56]. Post-isolation reactions can nonetheless perturb the membrane or aggregate vesicles, and the ligand density, accessibility and orientation per particle are harder to control than with genetic display.

#### 3.2.3. Membrane Fusion and Hybrid Vesicles

Membrane fusion joins the vesicle membrane to another that already carries the desired targeting or functional properties, transferring a whole repertoire of surface molecules at once rather than a single ligand. In a myocardial ischemia–reperfusion model, liposomes bearing the fibrin-targeting peptide CREKA were fused to the MSC membrane; the modified cells showed increased retention at the injury site and improved functional recovery [27]. A fusion-based display can therefore redirect cells to a target tissue, although whether it redirects their vesicles remains to be tested. Macrophage membranes have likewise been fused into umbilical cord blood MSCs and the hybrid cells were serially extruded into exosome-mimetic nanovesicles; the resulting nanovesicles carry integrin α4/β1 from the donor membrane, accumulate in the injured cord at twice the level of unmodified nanovesicles and improve locomotor recovery [28]. Most of the dose still reached the liver. Hybrids can also carry an engineered payload: macrophage and mitochondrial membranes fused together yield an artificial vesicle that homes to macrophages and carries a reactive oxygen species-activatable antioxidant prodrug, reprogramming these cells toward a reparative state in non-neural injury models [66]. Hybrid and biomimetic vesicles can combine homing, immune evasion and therapeutic cargoes in one particle at the cost of greater manufacturing complexity and a less defined composition.

#### 3.2.4. SCI-Relevant Targeting Ligands and Their Cellular Targets

The injured spinal cord contains cell populations that drive secondary damage and others that promote repair. Ligand selection therefore determines which populations interact with vesicles. Macrophages and microglia are the main targets of unmodified MSC-sEVs and the most extensively characterized therapeutic targets. Following infusion, MSC-sEV-associated signals are enriched in CD206-positive macrophages at the lesion site, described as M2 macrophages in previous reports [8,61]. Treated animals show a sustained shift toward an anti-inflammatory macrophage profile [9,61]. Although M2 macrophages are treated as a defined target in this review and elsewhere, the M1 response in the injured cord is sustained, whereas the M2 response is relatively small and transient [67]. The M1/M2 classification of microglia has also been questioned because it was adopted to aid interpretation before the relevant cell biology was characterized [68]. Vesicles directed toward this compartment therefore target a transient state rather than a stable cell population. This constrains administration timing and the interpretation of selective uptake. These cells are best described by their measured markers and functions. Unmodified MSC-sEVs reach these cells without engineered ligands. Studies of sEVs from other cell sources also inform cargo selection. In ischemic stroke, miR-124-enriched sEVs derived from M2 microglia reduce glial scar formation through the miR-124/STAT3 pathway in astrocytes [18]. This finding provides a non-MSC precedent for the cargo, but does not establish the same effect with an MSC-sEV carrier.

Engineered surface ligands reach the other cell populations selectively, and three are most pertinent to the injured cord, each incorporated by the genetic, chemical and fusion methods above. The rabies virus glycoprotein-derived peptide (RVG) targets neurons, a principal therapeutic target for restoring neurological function. RVG has been reported to bind nicotinic acetylcholine receptors on neuronal membranes and to direct vesicles to neurons and across the blood–brain barrier. However, whether receptor binding mediates barrier crossing remains unclear. RVG-displaying vesicles have delivered siRNA and small-molecule cargoes to the central nervous system (CNS) [21,23]. As a short peptide, RVG is compatible with all three attachment strategies and has been displayed on an MSC-sEV tested intravenously in the injured cord [24].

The Arg-Gly-Asp peptide (RGD) addresses the neurovascular compartment that governs barrier integrity. It binds to integrins, particularly αvβ3, that are upregulated on activated and neovascular endothelia. In an SCI model, RGD-engineered MSC-derived vesicles given intranasally targeted neovascular endothelial cells and were associated with enhanced angiogenesis, stabilization of the blood–spinal cord barrier and functional recovery [14]. A second RGD-displaying vesicle, derived from CD146+CD271+ umbilical cord MSC exosomes and also given intranasally, localized to the neovascular endothelium at the injured cord and repaired the blood–spinal cord barrier through the miR-501-5p/MLCK axis [25], which establishes RGD display on an MSC-sEV, not only on a synthetic carrier, as an achieved strategy for the cord. The same compartment includes pericytes, which MSC-derived vesicles protect from pyroptotic death, thereby preserving barrier function after injury [26]; whether an RGD-directed vesicle also reaches pericytes, or influences them only indirectly through endothelial stabilization, has not yet been tested. Neither RGD study in SCI quantified the fraction of the dose reaching the spinal cord or the magnitude of cellular uptake, and both administered the vesicles intranasally. Thus, these studies show RGD-mediated interaction with cells after the vesicles reach the spinal cord but do not determine the fraction of a systemically administered dose delivered to the cord (Section 3.3).

The Cys-Ala-Gln-Lys peptide (CAQK) addresses the lesion microenvironment itself rather than a cell type: it binds to chondroitin sulfate proteoglycan complexes that are upregulated in the injured brain and cord extracellular matrix; thus, it homes to the site of scarring, whereas a cell-directed ligand would not. Across the animal studies examined, CAQK conjugation improved lesion localization and, where functional outcomes were measured, recovery; additionally, CAQK-modified nanoparticles and liposomes accumulated at the spinal lesion to deliver metformin or rosuvastatin [29,30,69]. CAQK has also been carried on a vesicle: CAQK-modified, siRNA-loaded EVs from induced neural stem cells homed to the lesion and improved recovery after injury [31], a non-MSC precedent for the vehicle. CAQK is the most extensively studied lesion matrix-homing ligand in the cord, and it has been grafted onto a cargo-loaded MSC exosome tested there [32]. Most of the remaining evidence comes from synthetic nanoparticles, liposomes or non-MSC vesicles, and CAQK has not been compared directly with RVG or RGD under matched carrier, route, dose and model conditions.

No study of these three ligands measured the fraction of the administered dose that reached the cord. Evidence from other nanoparticle systems shows why this measurement matters. A pooled analysis of 232 datasets reported a median delivered fraction of 0.7% of the injected dose. Active targeting significantly improved delivery across all materials but not among organic carriers, the class most similar to EVs [70]. Thus, a relative targeting effect can be detected even when the absolute delivered fraction remains below 1%. Antibody-targeted and untargeted liposomes both accumulated in tumor tissue at 7–8% of the injected dose per gram. However, cancer cells internalized the targeted liposomes up to sixfold more efficiently than the untargeted liposomes, and the targeted liposomes produced greater therapeutic efficacy [71]. These findings show that ligand display can improve cellular uptake and therapeutic efficacy without increasing tissue accumulation. Tissue delivery and cellular uptake should therefore be measured separately.

Astrocytes and oligodendrocyte precursors complete the set of targets relevant to glial scarring and remyelination, although vesicle strategies aimed specifically at these cells remain less developed than those aimed at macrophages, neurons and the vasculature. The logic underlying all of them is the same: macrophages are reached by native homing, whereas neurons, the vasculature and the lesion matrix are reached by RVG, RGD and CAQK, respectively, so the ligand is chosen to match the cell or compartment the therapeutic goal requires.

### 3.3. Route and Retention

The route of administration determines the upper bound of how many vesicles reach the cord and how long they stay, and it interacts with every targeting strategy described above. Intravenous infusion is the least invasive route and the one that has been taken furthest, with use in patients in the parent MSC and MSC-sEV programs, but it exposes the dose to hepatic, splenic and pulmonary clearance, so only the homing and surface-targeting mechanisms preserve a meaningful lesion fraction [8,57]. Exosome preparations from ten sources all crossed the blood–brain barrier, although the crossing rates differed more than tenfold among sources. Inflammation altered uptake independently of barrier disruption [60]. However, intravenously administered untargeted EVs accumulated mainly in the liver, spleen, gastrointestinal tract and lungs [72]. In intact mice, intravenously administered MSC-EVs were detected mainly in the spleen and liver, while brain signals appeared only after intranasal delivery [58]. Whether SCI changes this route dependence remains unknown. Together, these findings suggest that peripheral clearance, rather than barrier entry alone, may limit delivery to the injured spinal cord.

Because a single bolus is cleared within hours, the dosing schedule also affects delivery. In chronic SCI, repeated weekly infusions of MSCs improved recovery more than a single infusion [73]. Continuous intravenous infusion of MSC-sEVs through an osmotic pump also produced greater therapeutic efficacy than separate injections of the same total dose, partly by sustaining the delivery of cargoes that remodel the lesion extracellular matrix [12].

Intrathecal delivery places vesicles directly into the cerebrospinal fluid and bypasses the systemic clearance that limits intravenous dosing, raising the concentration available to the cord. A phase I trial of intrathecal human umbilical cord MSC-derived vesicles in complete subacute injury found this route to be safe and feasible, thereby establishing a clinical foundation for the cerebrospinal fluid delivery of EVs [74]. The route is invasive, however, and distribution along the neuroaxis remains uncertain. Local delivery from an implanted depot addresses retention most directly: vesicles embedded in a hydrogel or scaffold at the lesion clear more slowly and release over days to weeks, with structural stability and bioactivity preserved [75,76], and engineered scaffolds deliver EVs locally without adverse immune reactions [77]. Pairing such depots with surface-targeted vesicles would combine persistence at the lesion with specificity of engagement.

Intranasal administration has been investigated as an alternative route for delivering MSC-sEVs to the CNS and was used in both RGD-targeted studies in SCI [14,25]. Labeled MSC exosomes accumulated more in the brain after intranasal administration than after intravenous administration [78]. However, the intranasal route does not eliminate systemic loss because substances crossing the nasal epithelium can enter the nasal vasculature. For proteins, less than 0.05% of the administered dose reaches the CNS [79]. Thus, intranasal administration may increase brain accumulation relative to intravenous administration, but absolute CNS delivery remains low.

## 4. The Payload: Equipping the Targeted Vesicle to Act

Targeting determines where vesicles accumulate and which cells they engage with; the payload determines what they deliver once they arrive. Targeting alone does not specify the mechanism or the potency of a designed product. Native vesicle cargo can be biologically active, but the heterogeneity of that cargo makes causal attribution and dose control difficult. For an engineered vesicle, the payload comprises two parts that are usually treated separately but should be specified together: the cargo molecule and the technology used to load it. The loading method constrains which cargoes are feasible and at what dose.

### 4.1. Cargo-Loading Technologies

Loading strategies fall into two families that differ in when the cargo enters the vesicle. Endogenous strategies engineer the parent cell so that secreted vesicles are pre-loaded, while exogenous strategies load the cargo into vesicles after isolation. The choice determines the trade-offs among loading efficiency, vesicle integrity, retained cargo function and manufacturing scalability that recur across the cargo classes below.

#### 4.1.1. Parent Cell (Endogenous) Loading

In endogenous loading, the parent cell is transfected or genetically engineered to overexpress the desired cargo, which is then sorted into vesicles through native packaging pathways and secreted in a functional form. Vesicles from MSCs transfected with an miR-133b mimic carry roughly 2.5-fold more miR-133b and, after intravenous injection, improve hindlimb functional recovery, reduce lesion volume and promote axonal regeneration in injured rats [80]. However, a strategy that increased microRNA loading into vesicles 65-fold did not deliver active microRNA to recipient cells [81]. Thus, studies must determine whether the loaded microRNA is functionally delivered to recipient cells.

Stable genetic engineering, including CRISPR-based editing of the parent cell, extends this approach from transient overexpression to durable modification of both the cargo and vesicle surface [82]. Endogenous loading preserves cargo folding and native packaging and pairs naturally with genetic surface display, but it is limited to cargoes that the cell will express and sort, and it requires control of the engineered cell line.

#### 4.1.2. Exogenous (Post-Isolation) Loading

Exogenous loading introduces cargo into already-isolated vesicles, which decouples the payload from parent cell biology and admits synthetic molecules and drugs. Electroporation transiently permeabilizes the membrane to drive in nucleic acids, and it was the method used to load siRNA into the first targeted, RVG-displaying vesicles [21]. Reported electroporation yields can overestimate siRNA loading. Electroporation without vesicles produced apparent siRNA retention equal to or greater than electroporation with vesicles because the electrical pulse precipitated insoluble siRNA aggregates that co-sedimented with the vesicle pellet. When aggregation was suppressed, retention fell below 0.05% and no longer differed between samples with and without vesicles [83].

Sonication, saponin permeabilization, freeze–thaw cycling and extrusion can load cargoes ranging from small molecules to proteins, but loading efficiency must be balanced against vesicle damage. Modifying the pH gradient across the vesicle membrane increased microRNA encapsulation compared with passive loading and preserved cargo function in recipient cells [84]. Other active methods can increase cargo loading but may disrupt membrane integrity, aggregate vesicles or co-isolate free cargo. Rigorous post-loading characterization is therefore required. Sonication, extrusion and saponin permeabilization differ in loading efficiency, release kinetics and preservation of cargo activity [85]. In a single-particle comparison of six strategies for loading a small hydrophobic drug, coincubation and electroporation achieved the highest encapsulation, whereas extrusion, freeze–thaw cycling, sonication and surfactant treatment damaged surface proteins [86]. A reported loading efficiency should therefore be interpreted as an upper bound when neither a no-vesicle control nor a functional readout in recipient cells is included, particularly for nucleic acid cargoes [87].

### 4.2. microRNA

microRNA is among the most extensively studied EV cargoes in SCI because a single short RNA can simultaneously repress many genes within a pathologic program. Several microRNA cargoes act by resolving inflammation: MSC vesicles delivering miR-146b suppress the TLR4/NF-κB axis to reduce inflammation and improve recovery [20], and microglial vesicles enriched in miR-124-3p shift recipient cells toward an anti-inflammatory state and promote neurite outgrowth after traumatic brain injury [16], with the same cargo from the same compartment subsequently shown to alleviate neurodegeneration and improve cognitive outcome after repetitive mild injury [17], a non-MSC precedent whose transfer to MSC-sEVs in the cord remains to be shown.

Other microRNA cargoes promote neuronal survival and regeneration. MSC vesicles carrying miR-26a-5p protect neurons by suppressing EZH2, thereby derepressing BDNF and activating TrkB-CREB signaling [22]. miR-133b-enriched MSC vesicles improve motor recovery in rats with SCI by suppressing RhoA and activating ERK1/2, STAT3 and CREB signaling [80]. MicroRNA cargoes may also remodel the lesion extracellular matrix (ECM). Continuous infusion of MSC-sEVs is associated with reduced production of scar-forming ECM at the lesion site. These vesicles are enriched in fibrosis-related microRNAs, suggesting that these cargoes contribute to ECM remodeling, although a direct causal link has not been established [12]. The range of reported effects expands engineering options but requires each microRNA cargo to be matched to the intended cellular target and phase of injury.

Attributing these effects to microRNA requires evidence that MSC-sEVs contain the proposed microRNA and deliver it to recipient cells at a functionally sufficient dose. Bulk assays do not provide this evidence. Because microRNAs are short and scarce, RNA extraction, reverse transcription, amplification and normalization can bias the measured abundance [15]. Non-vesicular microRNAs bound to proteins or other extracellular particles may also remain in the preparation [15]. Thus, bulk measurements do not establish intravesicular localization or copy number per vesicle. Across six exosome preparations from five sources, bulk RNA and particle counts indicated an average far below one copy of a given microRNA per vesicle [88]. A review of MSC exosomes estimated that 100 internalized vesicles would deliver about one copy, compared with an endogenous median of approximately 100 copies per cell, and proposed a predominantly protein-based mechanism [89]. Purified microRNA-containing vesicles bound to recipient cells but did not deliver detectable functional microRNA. Engineered fusion enabled messenger RNA delivery but produced no microRNA-dependent effect [90]. These findings show that detecting microRNA in an EV preparation does not establish functional delivery. Studies must therefore determine whether vesicle-associated microRNA reaches recipient cells and produces the reported therapeutic effect.

Evidence supporting functional microRNA delivery has also been reported in other systems. A reproducible microRNA-rich vesicle subpopulation was identified and shown to be functional [91]. Plasma EV fractions showed subpopulation-specific enrichment, although copy numbers were reported per sorted flow event rather than per vesicle [92]. Endogenous adipose-derived microRNAs also regulated gene expression in distant tissues [93]. However, no SCI study reviewed here combines single-vesicle quantification of the proposed microRNA with recipient-cell target engagement and a dose–outcome relationship. Thus, whether MSC-sEVs deliver sufficient microRNA to account for the reported effects remains unresolved [94]. This uncertainty does not negate the reported outcomes, but enrichment alone does not establish microRNA as the causal cargo. Attribution to microRNA therefore requires measured intravesicular loading, functional delivery to recipient cells and evidence that the delivered microRNA produces the reported therapeutic effect.

### 4.3. Proteins and Peptides

MSC−sEVs also deliver functional proteins, which act through receptor and signaling mechanisms distinct from the gene-level repression of microRNA. The anti-inflammatory protein TSG-6 is a recurring example: adipose-derived MSC-sEVs deliver TSG-6 to protect neurons and preserve the BSCB after spinal cord ischemia–reperfusion injury by inhibiting endoplasmic reticulum stress through the PI3K/AKT pathway, and TSG-6 carried by bone marrow stem cell vesicles attenuates neurotoxicity in a Parkinson’s model [33,95]. TSG-6 overexpression in the parent cells increased the motor recovery promoted by their secreted vesicles [33]. However, this result does not show whether TSG-6 carried by the vesicles or soluble TSG-6 released with them produced the effect. TSG-6 also mediates the effects of MSCs trapped in the lungs, even though the MSCs do not reach the lesion site [96]. Protein cargo is harder to load exogenously than nucleic acid and is most often delivered through endogenous packaging, which ties protein payloads to parent cell engineering and priming.

### 4.4. mRNA, Long Non-Coding RNA and siRNA

Beyond microRNA, vesicles carry longer and double-stranded RNA species that expand the range of achievable interventions. siRNA enables targeted knockdown of a single pathologic gene, and an electroporated MSC exosome preparation carrying CTGF-silencing siRNA reduced inflammation and glial scar formation and promoted locomotor recovery after injury [34]. No EV-free electroporation control was included, so the reported loading and contribution of encapsulated rather than co-isolated siRNA remain uncertain (Section 4.1.2). Long non-coding RNAs are an emerging cargo class implicated in the neuroprotective and immunomodulatory effects of MSC vesicles in central nervous system disease, although their mechanisms are less well defined than those of microRNA [97]. mRNA delivery, which transiently supplies a full protein-coding template, is established in other EV applications and remains comparatively underexplored in the cord. These cargoes are generally introduced via the exogenous loading of synthetic constructs or endogenous overexpression in the parent cell.

### 4.5. Exogenously Loaded Small-Molecule Drugs

Loading small-molecule drugs into vesicles combines a defined pharmacologic agent with the homing and barrier-crossing properties of the vesicle. RVG-displaying vesicles increased the brain accumulation and efficacy of cannabidiol compared with the free drug [23]. In the spinal cord, CAQK-targeted protein nanoparticles and liposomes delivered metformin and rosuvastatin, respectively, with sustained release at the lesion site [29,30]. These studies validated CAQK targeting and drug delivery in non-EV carriers. However, protein nanoparticles and liposomes are organic carriers, the class for which pooled tumor data showed no significant advantage of active targeting (Section 3.2.4). Neither study quantified the fraction of the injected dose delivered to the cord. Small-molecule cargoes can be loaded exogenously and combined with the surface strategies described in Section 3. The next study of a targeted MSC-sEV carrying a defined small-molecule cargo should report the fraction of the injected cargo dose that reaches the cord. This fraction can be measured directly.

## 5. Synthesis: Integrating Target, Cargo and Engineering Method

An engineered vesicle is defined by the combination of targeting and payload, which Section 3 and Section 4 treat separately. A given cellular target is best engaged by a particular surface strategy, and a given therapeutic goal dictates a particular cargo and loading method. An explicit matrix forces these choices to be specified together and shows where the field is mature and where inference comes from non-vesicle systems. Table 1 organizes representative studies along the three engineering axes that define a targeted vesicle: the cellular or molecular target, the delivered cargo, and the surface display strategy paired with its loading method (jointly, the engineering method). Each entry also records the parent cell, the mechanism, the model and the carrier context. Entries from non-MSC vesicles and non-vesicle systems each establish a targeting ligand, cargo or loading method transferable to MSC-sEVs, and serve as reference points rather than competing platforms. Reading the table by target rather than by study clarifies the present state of the field.

Three patterns emerge. First, the macrophage and microglial compartment is the most thoroughly characterized target and the one that native, unengineered vesicles engage with most selectively. MSC-sEVs delivered without surface modification already produce anti-inflammatory and vascular effects in rodent SCI studies [8,9], although some of those studies share investigators and independent replication with comparable products remains limited. Second, surface engineering can direct vesicles to targets that unmodified vesicles engage poorly. RVG directs vesicles to neurons, RGD to neovascular endothelial cells and CAQK to the ECM at the lesion site. The cargo is selected separately to produce the intended therapeutic effect at each target. Third, two MSC-sEV constructs combine an engineered targeting ligand with a deliberately loaded cargo. One is a CAQK-grafted hucMSC exosome carrying an electroporated CRISPR/Cas9 plasmid [32]. The other is an RVG- and TAT-displaying hucMSC exosome carrying a conjugated GPX4 activator [24]. Neither study reported ligand density per particle or the fraction of the injected dose that reached the cord. Neither included a comparison at a matched particle number. Thus, neither study establishes the added benefit of combining surface targeting with cargo loading. The same pairing has nonetheless been developed and tested on other platforms. CAQK-modified, siRNA-loaded vesicles from induced neural stem cells home to the lesion and improve recovery after SCI [31]; CAQK-bearing synthetic carriers deliver metformin and rosuvastatin to the lesion [29,30]; and RVG-displaying vesicles carry siRNA and a small-molecule drug into the CNS [21,23]. Together, these studies show that targeting ligands and defined cargoes can be combined on vesicular and synthetic carriers.The matrix therefore serves as a summary and design tool. For each therapeutic goal, it links the target to the surface strategy, cargo and loading method. It also distinguishes combinations supported by vesicle studies in the injured cord from those extrapolated from related systems. This comparison shows that the immediate priority is to characterize MSC-sEVs that already combine an engineered targeting ligand with a deliberately loaded cargo. Because the two components have been shown to function separately and have already been combined on this platform, the next step is to determine their individual and combined contributions to delivery and therapeutic efficacy.

## 6. Translational Status and Challenges

Engineering converts unmodified vesicles into designed delivery systems but also introduces new barriers to clinical translation. Early human studies provide preliminary safety data. Umbilical cord-derived EVs were used in a first-in-human study during spina bifida surgery [98], and intrathecal umbilical cord MSC-derived vesicles were reported as safe and feasible in a small uncontrolled phase I trial in patients with complete subacute SCI [74]. Neither study evaluated an engineered, systemically administered MSC-sEV or provided controlled evidence of efficacy. Experience with parent cell therapy also cautions against equating safety with efficacy. A phase III trial of intramedullary autologous MSC transplantation, a route distinct from intravenous infusion, showed limited efficacy in SCI [99]. Safety and biological plausibility do not ensure sufficient potency. Engineered MSC-sEVs must therefore be evaluated against defined efficacy criteria rather than safety alone.

### 6.1. Manufacturing, Potency and Characterization

Manufacturing and potency are the first challenges. Vesicle yield, cargo content and surface composition vary with parent cell source, passage and culture conditions, and the isolation method further alters the product; thus, dose and potency are difficult to define and reproduce across batches. The field has responded with consensus standards: MISEV2023 sets reporting and characterization requirements for EVs generally [15], and a position statement from the International Society for Extracellular Vesicles (ISEV) defines criteria specific to MSC-sEVs aimed at improving reproducibility and accelerating translation [36]. Engineering compounds this challenge because every modification adds an attribute that must be controlled and achieved in accordance with specifications. A genetically displayed ligand must be expressed at a consistent density, a chemically conjugated antibody must be coupled at a defined ratio without aggregating the vesicle, and an exogenously loaded cargo must be quantified and distinguished from free, co-isolated molecules. Characterization of engineered EVs therefore expands beyond identity and purity to include ligand density, loading efficiency and the retained function of both the surface and the cargo.

### 6.2. Scalable, GMP-Compatible Engineering

The second challenge is to make the engineering strategy scalable and compatible with good manufacturing practice. Endogenous strategies for engineering parent cells, including stable genetic and CRISPR-based modifications, produce vesicles from a uniformly modified cell population and fit within established cell culture workflows, but they require a controlled, well-characterized engineered cell line [82]. Exogenous strategies decouple the payload from the cell and admit synthetic drugs, yet methods such as electroporation and conjugation chemistry can perturb the vesicle and remain difficult to standardize [56]. Precision and ease of manufacture therefore trade off against each other.

### 6.3. Dosing and Source Variability

Dose, schedule and timing remain empirical. Continuous infusion of MSC-sEVs outperformed separate injections at the same total dose [12]. Repeated whole-MSC infusion also improved outcomes [73], but this finding does not separate schedule from cumulative exposure and is not direct EV evidence. A network meta-analysis suggested that EVs derived from microRNA-overexpressing MSCs may have greater early potency per dose but did not establish a greater final extent of recovery [100]. The optimal route, dose and timing remain undetermined and may differ between surface-targeted and depot-released vesicles.

Dose must also be controlled when products are compared. In transcriptomic studies, dose affected recipient-cell responses more than source [101]. These experiments were mainly performed in vitro, with in vivo single-cell RNA-seq confirmation, and did not assess locomotor efficacy. Engineered and unmodified vesicles should therefore be compared at matched particle numbers to avoid attributing dose effects to engineering.

Products from different donors or tissues may also differ in potency. Tissue of origin alone therefore cannot establish product equivalence.

### 6.4. What the Field Has Not Established

Available evidence does not establish that engineering itself improves therapeutic efficacy. One meta-analysis reported improved locomotor scores after stem cell-derived EV treatment in acute SCI, but funnel plot asymmetry indicated possible publication bias, heterogeneity or other small-study effects [40]. A larger analysis found numerically greater improvement with engineered than unmodified vesicles, but did not compare the groups directly [102]. Because both analyses pooled products that differed in source and preparation, their results apply to heterogeneous preparations rather than to a defined product.

Transferability is also uncertain. Much of the evidence for CAQK targeting comes from synthetic nanoparticles or non-MSC vesicles [69]. Whether these results extend to engineered MSC-sEVs has not been established.

The site of therapeutic action remains uncertain. Detection of MSC-sEV-associated signals in lesion macrophages supports a local mechanism [8,9], but systemic effects may also contribute. In other disease models, lung-trapped MSCs acted through secreted TSG-6 [96], stem cells accumulated in the spleen but reduced cerebral injury and inflammation [103] and MSC-EVs improved stroke recovery by altering peripheral rather than cerebral immune populations [104]. These non-SCI findings raise the possibility that lesion targeting may not improve and could reduce systemic therapeutic effects. Greater lesion delivery therefore cannot be assumed to improve therapeutic efficacy.

### 6.5. What to Adopt from Adjacent Fields

These gaps require quantitative measurements already used in adjacent fields. Tumor nanomedicine addressed a similar delivery problem (Section 3.2.4). SCI studies should report the fraction of the injected dose that reaches the cord together with a blood-normalized exposure ratio because the dose fraction alone correlates poorly with standard pharmacokinetic measures [105]. Cell-specific uptake should be reported separately from bulk tissue accumulation.

The bio–nano minimum-reporting standard requires ligand number and affinity, label intensity per particle in absolute units and size and charge in the relevant biological medium [106]. Applying this standard to engineered MSC-sEVs would replace qualitative claims of ligand display with quantitative particle characterization and make labeling artifacts easier to detect (Section 3.1).

Cell and gene therapies are qualified by potency assays rather than by starting material alone. Recommendations for MSC-sEV potency testing apply the same principle [37], and a societal synthesis identifies the context-specific active ingredient as a priority [38]. Studies should report particle number and tissue of origin and assess engineered MSC-sEVs with a mechanism-linked potency assay. Reporting these measures together would allow comparisons across batches, donors and sources and support batch release against preset specifications. Together, these standards require quantitative measures of target delivery, particle cargo and product potency.

## 7. Future Directions

The next step is to determine whether combining surface targeting with cargo loading improves therapeutic efficacy. A CAQK-modified MSC-sEV carrying a cargo previously tested in SCI should be compared with an unmodified MSC-sEV and a scrambled peptide control at the same particle dose and time points. The study should measure delivery to the lesion, cargo uptake by recipient cells, locomotor recovery and peripheral immune responses. Each vesicle preparation should also be characterized using the standards described in Section 6.5 and tested with a mechanism-linked potency assay [37]. This direct comparison is needed because the available meta-analysis compared engineered and unmodified vesicles only indirectly [100].

## 8. Conclusions

Unmodified MSC-sEVs are associated with improved locomotor outcomes in rodent SCI models, but product heterogeneity and risk of bias limit conclusions for a defined product. After systemic administration, only a small fraction of the dose reaches the injured cord, and recipient-cell uptake is not controlled. Surface engineering is intended to increase delivery to the cord and control recipient-cell engagement. Two reports found preferential neovascular localization and improved barrier function after intranasal administration of RGD-engineered umbilical cord MSC-sEVs. However, the fraction of the dose reaching the cord and the efficiency of intravenous targeting remain unknown. Two MSC-sEV constructs have combined an engineered ligand with a deliberately loaded cargo, and similar designs have been tested with non-MSC vesicles and synthetic carriers. These studies establish feasibility but do not show how much targeting and cargo loading each contributed to delivery or therapeutic efficacy.

Three questions remain: whether MSC-sEVs from different tissues are equivalent, whether they deliver sufficient amounts of the proposed cargoes to account for the reported effects and whether the therapeutic effects of systemically administered MSC-sEVs arise mainly within the cord or partly through peripheral immunomodulation. Resolving these questions is necessary to select the vesicle source, target and cargo for an engineered MSC-sEV.

## Figures and Tables

**Table 1:** Target, cargo and engineering method for MSC-sEV-based delivery to the injured spinal cord. The parent cell (source) is listed for every entry to separate MSC-derived vesicles, the focus of this review, from other source vesicles and non-vesicle systems that are retained as examples of transferable methodology. The surface strategy and loading method are tagged per study; the model and carrier context column distinguishes MSC-sEV studies in SCI models from methods demonstrated in other cells (non-MSC), non-EV carriers (synthetic nanoparticles or liposomes) and whole-cell models or by non-vesicle transfer (tunneling nanotube or isolated organelle). Such non-MSC or non-EV entries establish a ligand, cargo or loading method that can be transferred to MSC-sEVs. A cargo is classified as deliberately loaded when a defined agent is intentionally introduced into the vesicle. Entry [14] modifies the parent cell state without selecting a cargo and is therefore classified as a surface strategy. The EV type column records the term that each original study used for its own preparation. Most used the term exosome without demonstrating endosomal origin, which MISEV2023 discourages [15]. The original term is therefore retained rather than reassigned, as size and a limited marker panel do not by themselves establish sample identity, and the column is included so that this terminological variation and the presence of non-vesicle carriers among the entries remain visible.

Target	Parent Cell (Source)	EV Type (as Described in Original Study)	Cargo	Surface Strategy	Loading Method	Mechanism/Outcome	Model and Carrier Context	Ref
CD206+ macrophages	MSC (bone marrow)	Exosomes	Native MSC cargo	Native accumulation	Native packaging	Preferential association with CD206+ lesion macrophages; no therapeutic endpoint reported	In vivo, SCI (biodistribution)	[8]
CD206+ macrophages	MSC (bone marrow)	sEVs	Native MSC cargo	Native accumulation	Native packaging	Anti-inflammatory shift, TGF-β upregulation, microvascular stabilization, motor recovery	In vivo, SCI	[9]
Macrophages/microglia	Microglia (BV2)	Exosomes	miR-124-3p	Native (microglial sEV)	Native packaging	Anti-inflammatory effect on neurons, neurite outgrowth	In vivo, TBI (non-MSC)	[16]
Neurons/neurodegeneration	Microglia (BV2)	Exosomes	miR-124-3p	Native (microglial sEV)	Parental-cell mimic transfection	Reduced neurodegeneration and improved cognition via neuronal Rela-ApoE	In vivo, repetitive mild TBI (non-MSC)	[17]
Microglia/glial scar	M2 microglia (BV2)	sEVs	miR-124	Native (microglial sEV)	Native packaging	Reduced glial scar via miR-124/STAT3	In vivo, stroke (non-MSC)	[18]
Neutrophils/lesion	MSC (amnion)	Exosomes	miR-125a-3p	Native accumulation	Native (unmodified)	Suppressed NET formation	In vivo, SCI	[19]
Lesion inflammation	MSC (umbilical cord)	Exosomes	miR-146b	Native accumulation	Native packaging	TLR4/NF-κB suppression, improved recovery	In vivo, SCI	[20]
Neurons	Dendritic cell	Exosomes	siRNA	RVG-Lamp2b (genetic)	Electroporation	Specific, non-immunogenic knockdown across BBB	In vivo, brain (non-MSC origin)	[21]
Neurons	MSC (bone marrow)	Exosomes	miR-26a-5p	Native accumulation	Native packaging	Neuroprotection via EZH2/BDNF-TrkB-CREB	In vivo, SCI	[22]
Brain/CNS (cellular target not established)	HEK293T	Exosomes	Cannabidiol	RVG (genetic)	Exogenous loading	Increased brain accumulation vs. free drug	In vivo, CNS (non-MSC)	[23]
Neurons	MSC (umbilical cord)	Exosomes	GPX4 activator (small molecule)	RVG and TAT via CP05 anchor (post-isolation)	Chemical conjugation (NHS ester)	Ferroptosis inhibition, axon regeneration, functional recovery	In vivo, SCI (MSC exosome)	[24]
Neovascular endothelium	MSC (umbilical cord, endothelial-like differentiated)	Exosomes	Native E-UCMSC cargo	RGD (display)	Parent-cell differentiation (state change, not cargo selection)	Angiogenesis, BSCB stabilization	In vivo, SCI (intranasal)	[14]
Neovascular endothelium	MSC (umbilical cord, CD146+CD271+)	Exosomes	miR-501-5p (native)	RGD (genetic display)	Native packaging	BSCB repair via miR-501-5p/MLCK	In vivo, SCI (intranasal)	[25]
Pericytes	MSC (bone marrow)	Exosomes	Native MSC cargo	Native accumulation	Native packaging	Protection from pyroptosis, barrier preservation	In vivo, SCI	[26]
Lesion ECM	MSC (bone marrow)	sEVs	Native sEV (fibrosis-related miRNA)	Native accumulation	Native packaging	Reduced scar-forming ECM on continuous infusion (associative)	In vivo, SCI	[12]
Lesion ECM (fibrin)	MSC (whole cell)	Not an EV study (whole cells)	Cell-based model	CREKA-liposome membrane fusion	Not applicable (whole-cell modification)	Increased lesion retention, functional recovery	In vivo, myocardial (cell-based, non-EV)	[27]
Injured cord	MSC (umbilical cord blood)	Exosome-mimetic nanovesicles (extruded)	Native cargo	Macrophage-membrane hybrid (fusion)	Extrusion (nanovesicle)	Donor-membrane homing, lesion treatment	In vivo, SCI (engineered nanovesicle)	[28]
Lesion ECM	Synthetic (none)	Non-EV: polymeric nanoparticle	Metformin	CAQK	Exogenous (non-EV)	Lesion-targeted delivery, improved recovery	In vivo, SCI (non-EV nanoparticle)	[29]
Lesion ECM	Synthetic (none)	Non-EV: liposome	Rosuvastatin	CAQK	Exogenous (non-EV)	Sustained lesion delivery, improved recovery	In vivo, SCI (non-EV liposome)	[30]
Lesion ECM	iNSC (non-MSC)	EVs	CCL2-siRNA	CAQK (chemical)	Electroporation	Lesion-targeted delivery, M1-to-M2 shift, recovery	In vivo, SCI (non-MSC EV)	[31]
Lesion ECM/macrophages	MSC (umbilical cord)	Exosomes	CRISPR/Cas9 plasmid (sTNFR1 circuit)	CAQK (chemical, EDC/NHS)	Electroporation (post-isolation)	Lesion accumulation, macrophage reprogramming, BMS recovery	In vivo, SCI (MSC exosome)	[32]
Neurons/BSCB	MSC (adipose)	sEVs	TSG-6 (protein)	Native accumulation	Parent-cell engineering	ER-stress inhibition via PI3K/AKT, barrier protection	In vivo, SCI-IR	[33]
Glial scar	MSC (bone marrow)	Exosomes	CTGF-silencing siRNA	Native accumulation	Exogenous loading	Reduced inflammation and scar, locomotor recovery	In vivo, SCI	[34]

## Data Availability

No new data were created or analyzed in this study. Data sharing is not applicable to this article.
